# Adipose-derived stem cell exosomes regulate Nrf2/Keap1 in diabetic nephropathy by targeting FAM129B

**DOI:** 10.1186/s13098-023-01119-5

**Published:** 2023-07-04

**Authors:** Peiyao Ren, Fengmei Qian, Lanjun Fu, Wenfang He, Qiang He, Juan Jin, Danna Zheng

**Affiliations:** 1 The Second School of Clinical Medicine, Zhejiang Chinese Medical University, Hangzhou, Zhejiang 310003 China; 2 Department of Nephrology, the First Affiliated Hospital of Zhejiang Chinese Medical University, Zhejiang Provincial Hospital of Traditional Chinese Medicine), Hangzhou, Zhejiang 310000 China; 3 Urology & Nephrology Center, Department of Nephrology, Affiliated People’s Hospital, Zhejiang Provincial People’s Hospital, Hangzhou Medical College, Hangzhou, Zhejiang 310014 China

**Keywords:** Adipose-derived stem cells, Exosomes, Diabetic nephropathy, Oxidative stress, Inflammation, Nrf2/Keap1 pathway

## Abstract

**Background:**

Exosomes from adipose-derived stem cells (ADSCs-Exos) have exhibited a therapeutic role in diabetic nephropathy (DN). Further studies are needed to investigate how ADSCs-Exos regulate oxidative stress and inflammation in high glucose-induced podocyte injury.

**Methods:**

An enzyme-linked immunosorbent assay (ELISA) was used to detect cellular inflammation. Reactive oxygen species (ROS) levels were assessed using flow cytometry in podocytes with different treatments. A malondialdehyde (MDA) kit was used to evaluate the lipid peroxidation levels in podocytes and kidney tissues of mice. Western blotting and co-immunoprecipitation were performed to detect protein expression and protein-protein interactions.

**Results:**

ADSCs-Exos reversed oxidative stress and inflammation in podocytes and kidney tissues of DN mice induced by high glucose levels in vitro and in vivo. Interference with heme oxygenase-1 expression could reverse the improvement effect of ADSCs-Exos on oxidative stress induced by high glucose levels. Furthermore, high glucose inhibited nuclear factor erythroid 2-related factor 2 (Nrf2) protein expression and promoted Kelch-like ECH-associated protein 1 (Keap1) protein expression in podocytes, as well as their binding ability. As a potential target for Nrf2/Keap1 pathway regulation, FAM129B expression in podocytes is regulated by high glucose and ADSCs-Exos. Moreover, FAM129B siRNA blocked the inhibitory effect of ADSCs-Exos on intracellular ROS and MDA upregulation induced by high glucose in podocytes.

**Conclusion:**

ADSCs-Exos regulate the Nrf2/Keap1 pathway to alleviate inflammation and oxidative stress in DN by targeting FAM129B, which may provide a potential therapeutic strategy for DN.

**Supplementary Information:**

The online version contains supplementary material available at 10.1186/s13098-023-01119-5.

## Introduction

Diabetic nephropathy (DN) is a common complication of diabetes and is recognized as the leading cause of end-stage kidney disease [1]. Up to one-third of patients with diabetes are affected by impaired renal function, leading to poor prognosis and a heavy social and economic burden over time [2].

Mesenchymal stem cells (MSCs) have received increasing attention in recent years for their promising anti-inflammation and immunomodulatory functions [3, 4]. Adipose-derived stem cells (ADSCs), which can be easily harvested from various tissues, are multipotent and can secrete bioactive factors, such as proteins and RNA [5]. These factors are often secreted in the form of extracellular vesicles—including exosomes (Exo) which are nanoscale vesicle 30–150 nm in size—and transported to various parts of the body through autocrine, endocrine, and paracrine pathways to play a role in regulating tissue homeostasis [6, 7]. Consequently, exosomes can serve as biomarkers and therapeutics for various kidney disorders [8, 9]. ADSCs-Exo have become a subject of interest in DN therapy in recent years [10]. We also found that ADSCs-Exo ameliorated the HG-stimulated podocytes and DN albuminuria progression [11]. However, the specific mechanisms remain unclear and require further research.

Current researches have provided evidence that inflammatory responses and oxidative stress play crucial roles in DN progression [12–14], leading to increased proteinuria, glomerular endothelial cell damage, and renal fibrosis, triggering the onset of DN [15]. Interestingly, MSC-derived exosomes have been found to exert anti-inflammatory and anti-oxidative functions in a variety of diseases. [16, 17].

The Kelch-like ECH-associated protein 1 (Keap1)-nuclear factor erythroid 2-related factor 2 (Nrf2)-antioxidant response element (ARE) pathway is a classical antioxidant stress pathway that mediates the transcriptional regulation of various antioxidant factors [18, 19]. Physiologically, Nrf2 can be trapped by Keap1 and degraded through the proteasome pathway to maintain a low levels [20]. Under stress, Nrf2 dissociates from Keap1 and is transported to the nucleus to specifically regulate ARE transcription [21]. As an anti-inflammatory, proliferative, angiogenic, and cytoprotective enzyme, Heme oxygenase-1 (HO-1) is an influential downstream factor of this pathway, exerting antioxidant effects [22] which can heal diabetic foot wounds [23].This pathway has a potential role in reversing high glucose (HG)-induced podocyte injury, although the specific mechanism is not fully understood.

Therefore, we investigated the mechanism underlying ADSC-Exo function in DN progression. We demonstrated that ADSCs-Exos can reduce oxidative stress and inflammation in podocytes via the Keap1/Nrf2/ARE pathway. This lays the groundwork for further clinical applications of ADSC-Exo.

## Materials and methods

### Exosome isolation and identification

Exosome extraction and identification were performed as previously described [11, 24]. The exosomes used in this study were extracted by our research group and frozen in liquid nitrogen for long-term use.

### Cell culture and treatment

MPC5 mouse podocytes were obtained from icell Bioscience (iCell-m081, Shanghai, China). The cells were seeded into culture flaps coated with type I collagen (Gibco,17100-017) and amplified in RPMI-1640 medium (Hyclone, SH30809.01b) with 20 U/mL IFN-γ (GMP-TL105) and 10% fetal bovine serum (Gibco, San Diego, CA, USA) at 33 °C. MPC5 cells were differentiated into mature podocytes after incubation in RPMI-1640 medium without IFN-γ but containing 5% FBS at 37 °C for 14 days. MPC5 cells were cultured in low-glucose (5.5 mM D-glucose), HG (30 mM D-glucose), or hypertonic [5.5 mM D-glucose + 24.5 mM mannitol (MA)] medium, and treated with solvents or 25 µg/mL ADSCs-Exos combined with NC siRNA or HO-1 siRNA or FAM129B siRNA as designed for 72 h after 24 h of synchronous culture in RPMI-1640 medium with 0.2% FBS. The siRNA sequences are listed in Supplementary Table 1.

### Animal treatment

C57BL/KsJ db/m (control mice, n = 10) and C57BL/KsJ db/db (spontaneous diabetes mice, n = 20) male mice were housed in a 12 h light/dark cycle under constant temperature and humidity until 12-weeks-old. After identification via periodic acid-Schiff staining, PBS or ADSCs-Exos (100 µg/mL) were injected into mice from each group through the tail vein. After 12 weeks, the mice were euthanized, and kidney tissues were removed and stored in liquid nitrogen for subsequent experiments.

### Immunofluorescence

The cells were fixed with 4% paraformaldehyde for 15 min and permeabilized with 0.5% Triton X-100 for 20 min at room temperature. The cells were blocked with goat serum for 30 min at room temperature. Then they were incubated with the primary antibody at 4 °C overnight. This was followed by a wash with PBST, following which they were incubated at 37 °C for 1 h with a fluorescence-labeled secondary antibody (BOSTER, Wuhan, China, BA1032; 1:500). Then, the cells were stained with DAPI (Beyotime, Shanghai, China, C1002) for 5 min in the dark. Images were observed under a fluorescence microscope (Olympus, BX53) after the mounting the cells in anti-fade mounting medium (Southernbiotech, 0100-01).

### Western blotting

Radioimmunoprecipitation assay buffer (Cell Signaling Technology) was mixed with phosphatase and protease inhibitors to lyse the cells, and total protein was obtained by centrifugation. Samples were quantified with a BCA kit (Beyotime, Shanghai, China, P0012S), electrophoresed on 10% SDS-PAGE gels, and transferred to a polyvinylidene fluoride membrane according to their molecular weight. After blocking with 5% milk, the membrane was incubated with primary antibodies at 4 °C. Secondary antibodies were incubated with the membranes for 1 h at room temperature. An electrochemiluminescence kit was used to visualize the chemiluminescence.

### Co-immunoprecipitation (Co-IP)

After lysis with IP lysate (Beyotime, Shanghai, China; P0013) for 15 min on ice, the supernatant was collected via centrifugation. The samples were pretreated with agarose A + G and slowly shaken at 4 °C for 2 h. IgG or Keap1antibodies were added to 500 µL of total protein. The antigen-antibody mixture was slowly shaken overnight at 4 °C. Then, 30 µL of 50% agarose protein A + G was added to each tube, and the reaction was carried out for 6 h at 4 °C. After instantaneous high-speed centrifugation, the precipitate was collected and washed with precooled PBS. Then, 2× loading buffer was added to the precipitate and resuspended, followed by a metal bath reaction at 100 °C for 5 min and immediate cooling on ice. The supernatant was collected via centrifugation at 12,000 rpm for 10 min. The input group without IgG or Keap1 antibodies was used as a control. Protein samples from the input, anti-IgG, and anti-Keap1 groups were subjected to western blotting to detect Keap1 and Nrf2 levels.

### Enzyme-linked immunosorbent assay (ELISA)

A double antibody sandwich was used to detect the levels of IL-1β (Beyotime, Shanghai, China; PI301), IL-6 (Beyotime, Shanghai, China; PI326), and TNF-α (Beyotime, Shanghai, China; PT512). Samples or different standard concentrations were added to a 96-well plate and incubated for 120 min at room temperature. Afterwards, each well was incubated for 60 min at room temperature with 100 µL biotinylated antibody. The plate was then incubated in the dark for 20 min at room temperature with 100 µL of horseradish peroxidase-labeled streptavidin per well. The A_450_ value was determined immediately after mixing the TMB solution with the termination solution at room temperature in the dark for 20 min.

### Reactive oxygen species (ROS) assay

First, 2 × 10^5^ cells were seeded in 12-well plates and cultured overnight at 37 °C in a 5% CO_2_ incubator. Diluted DCFH solution (Beyotime, S0033S-1) was added after the cells were washed with PBS and collected via centrifugation. The mixture was mixed every 3 min for 21 min of incubation at 37 °C, and the dyes were removed using serum-free medium after incubation. The resuspended cells were stimulated with ROS-positive control (Beyotime, S0033S-2) for 20 min. Subsequently, flow cytometry was performed.

### Malondialdehyde (MDA) assay

The MDA content was detected using the TBA method (Nanjing Jiancheng Bioengineering Institute, A003-1). Following the addition of the kit extract to the sample, the working solution was added, and the supernatant was collected after 40 min in a water bath at 95 °C. Absorbance was measured at 532 nm. The standard substance detection results were used to calculate MDA concentrations.

### Statistical analysis

Prism 9.0 was used to statistically analyze the data. All data are presented as means ± standard deviation (SD). One-way analysis of variance was used for statistical analysis between the groups. Differences were considered significant at *P* < 0.05.

## Results

### ADSCs-Exos alleviate HG-induced inflammation and oxidative stress in podocytes

**Fig. 1:**
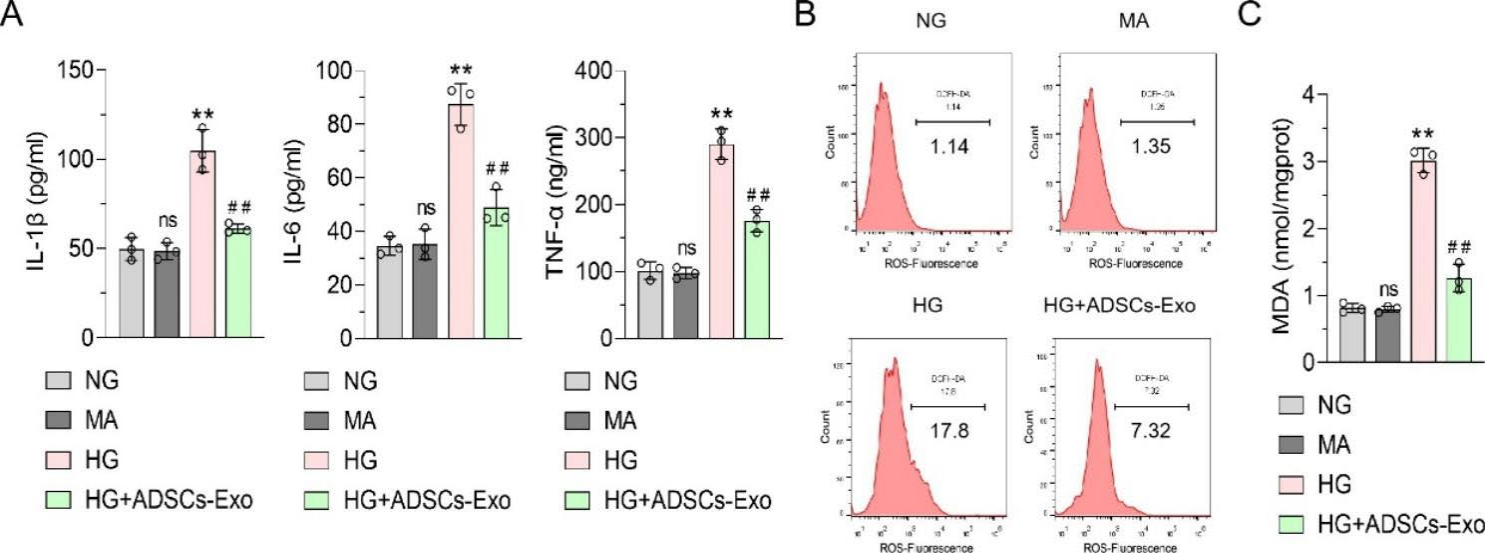
**Effects of high glucose and ADSCs-Exo on podocyte inflammatory cytokines secretion and oxidative stress** **(A)** ELISA was used to detect the levels of inflammatory cytokines IL-1β, IL-6 and TNF-α in the supernatant of MPC5 cells in each group. **(B)** Flow cytometry was used to detect the level of ROS in MPC5 cells. **(C)** MDA kit was used to detect MDA content in MPC5 cells of each group. Data were presented as Mean ± SD and one-way analysis of variance was used to detect statistical differences between groups, ns p > 0.05 VS. NG; ** p < 0.01 VS. NG; ## p < 0.01 VS. HG.

To determine the inflammatory and oxidative stress status of podocytes under diabetic condition, 30 mM D-glucose medium was used to mimic diabetic states in vitro, while 5 mM D-glucose medium was used to mimic normal states in vitro. ELISA showed that the secretion of the inflammatory cytokines IL-1β, IL-6, and TNF-α in podocytes under HG conditions was significantly higher than that under low glucose conditions. MA was used to simulate the hypertonic state of cells to exclude the effect of osmotic pressure. The secretion levels of IL-1β, IL-6, and TNF-α in MPC5 cells in the MA group were not significantly different from those in the normal glucose (NG) group, which showed that a HG, but not hypertonic environment could increase the secretion of proinflammatory factors in podocytes (Fig. 1A). Subsequently, the ROS in MPC5 cells in each treatment group were analyzed using flow cytometry, and the ROS increased in the HG group, compared with that in the NG group (Fig. 1B). MDA quantitative detection showed similar results (Fig. 1C). Moreover, ROS and MDA levels in MPC5 cells in the MA group did not change, compared with those in the NG group, which confirmed that oxidative stress in podocytes was indeed mediated by HG, rather than a hypertonic environment. Further, we used ADSCs-Exos to interfere with MPC5 cells to clarify their therapeutic effect on HG-induced podocytes. We found that the secretion of IL-1β, IL-6, and TNF-α, as well as ROS and MDA levels in MPC5 cells in the ADSC-Exo group were significantly decreased, compared with those in the HG group (Fig. 1A and C). The results demonstrated that ADSCs-Exos could reverse HG-induced podocyte oxidative stress and proinflammatory factor secretion.

**Fig. 2:**
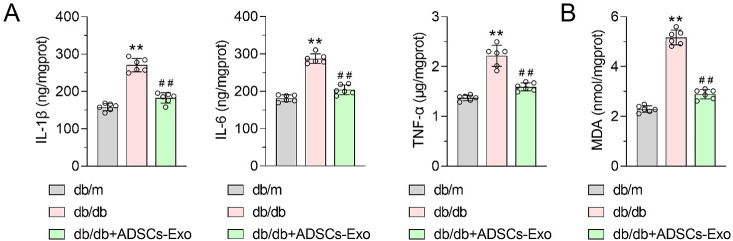
**Effect of ADSCs-Exo on the content of inflammatory cytokines and MDA in renal tissues of mice with diabetic nephropathy** **(A)** ELISA was used to detect the levels of inflammatory cytokines IL-1β, IL-6 and TNF-α in kidney tissue of mice in each group. **(B)** MDA kit was used to detect MDA content in kidney tissue of mice in each group. Data were presented as Mean ± SD, and one-way analysis of variance was used to detect statistical differences between groups, ** p < 0.01 VS. db/m; ## p < 0.01 VS. db/db

These in vivo results were consistent with the in vitro results. Compared with db/m mice, IL-1β, IL-6, TNF-α, and MDA levels increased significantly in the kidney tissues of db/db mice and decreased significantly in ADSC-Exo-treated DN mice (Fig. 2A and B). ADSCs-Exos could reverse the increase in inflammatory cytokines IL-1β, IL-6, TNF-α, and MDA levels in the renal tissues of DN mice.

### ADSCs-Exos target HO-1 to reverse elevated podocyte inflammation and oxidative stress levels induced by HG

**Fig. 3:**
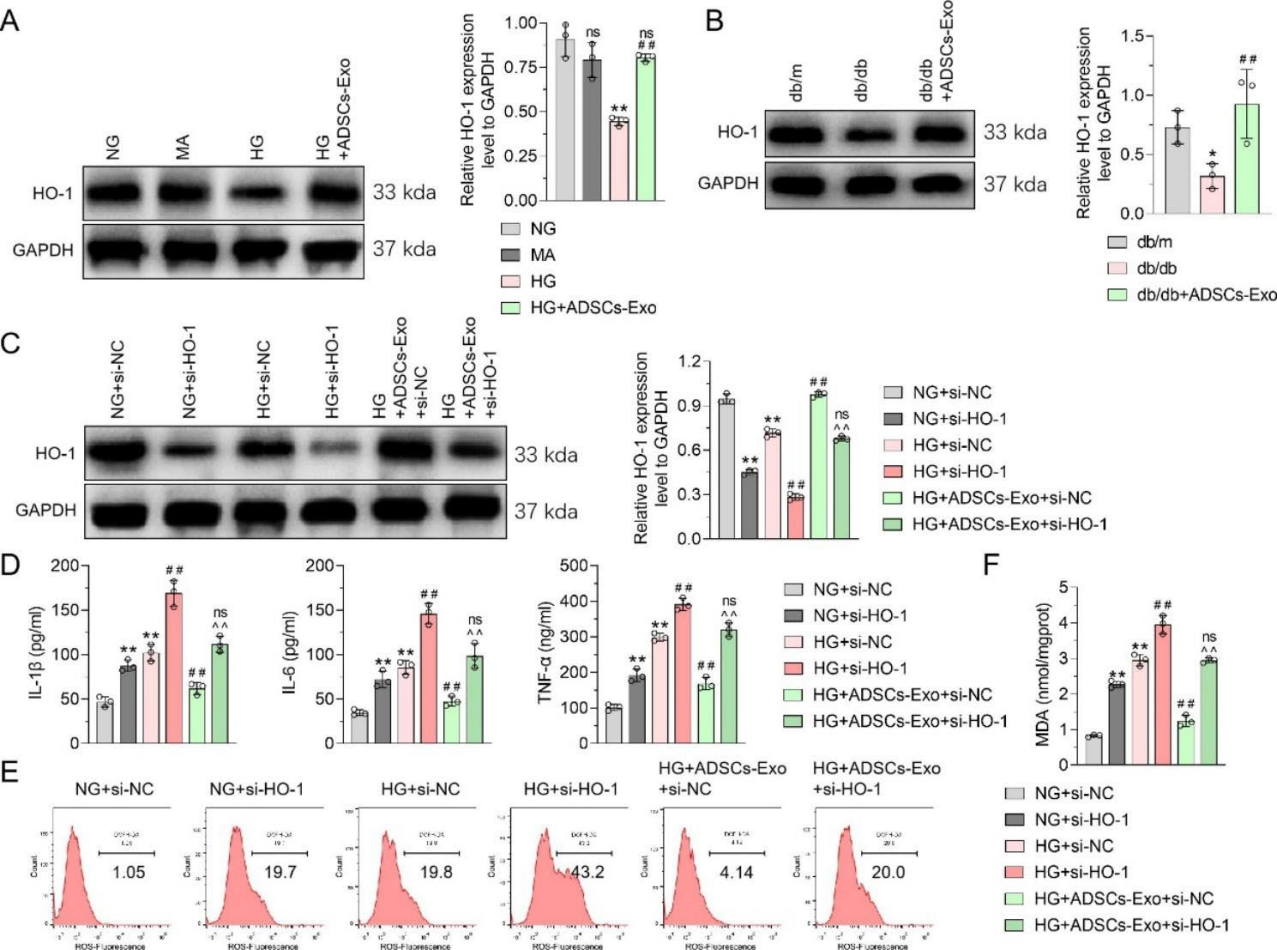
**Effect of HO-1 siRNA and ADSCs-Exo on the secretion of inflammatory cytokines and oxidative stress in podocytes under high glucose treatment** **(A)** WB was used to detect the effect of ADSCs-Exo on HO-1 expression in MPC5 cells under high glucose treatment, and GAPDH was used as an internal control. **(B)** WB was used to detect the effect of ADSCs-Exo on HO-1 expression in kidney tissue of model mice, and GAPDH was used as an internal control. **(C)** WB was used to detect the effect of HO-1 siRNA and ADSCs-Exo on HO-1 expression in MPC5 cells under high glucose treatment, with GAPDH as an internal control. **(D)** ELISA was used to detect the effects of HO-1 siRNA and ADSCs-Exo on the secretion of pro-inflammatory factors in MPC5 cells treated with high glucose. **(E)** Flow cytometry was used to detect the effects of HO-1 siRNA and ADSCs-Exo on ROS content in MPC5 cells under high glucose treatment. **(F)** The effect of HO-1 siRNA and ADSCs-Exo on MDA content of MPC5 cells treated with high glucose was detected by MDA kit. Data are presented as Mean ± SD and one-way analysis of variance was used to detect statistical differences between groups. Panel A, ns p > 0.05 VS. NG; ** p < 0.01 VS. NG; ## p < 0.01 VS. HG. Panel B, * p < 0.05 VS. db/m; ## p < 0.01 VS. db/db. Panel C, D, F, ** p < 0.01 VS. NG + si-NC; ns p < 0.05, ## p < 0.01 VS. HG + si-NC; ^^ p < 0.01 VS. HG + ADSCs-Exo + si-NC.

The expression of HO-1, an important antioxidant gene, was significantly decreased in the HG group compared to its level in the NG and MA groups. Further, ADSCs-Exos reversed the glucose-induced decrease in HO-1 levels (Fig. 3A). Similar results were observed in vivo; HO-1 expression in the kidney tissue of DN mice was significantly reduced, compared with that in db/m mice, but it recovered after ADSCs-Exos treatment, indicating that ADSCs-Exos reversed the HG-induced decrease in HO-1 in podocytes and DN renal tissue (Fig. 3B).

To explore the role of HO-1 in the protection of ADSCs-Exos-stimulated podocytes, HO-1 siRNA was found to significantly reduce the HO-1 content in MPC5 cells. Si-HO-1-2 was selected for further studies (Supplementary Fig. 1). HO-1 siRNA increased the glucose-mediated inhibition of HO-1 expression. Further, HO-1 siRNA reversed the inhibitory effect of ADSCs-Exos on decreased HO-1 expression in HG-stimulated MPC5 cells (Fig. 3C). Subsequently, the effects of HO-1 siRNA and ADSCs-Exos on IL-1β, IL-6, and TNF-α secretion in MPC5 cells treated with HG were investigated using ELISA. These findings demonstrated that HO-1 siRNA not only increased the levels of IL-1β, IL-6, and TNF-α, but also enhanced HG-mediated proinflammatory responses. Moreover, HO-1 knock-down could block the ADSCs-Exos inhibition of IL-1β, IL-6, and TNF-α secretion in HG-treated MPC5 cells. (Fig. 3D). Flow cytometry and MDA detection showed that HO-1 siRNA increased the ROS content and MDA levels in MPC5 cells, regardless of whether the cells were treated with HG. Furthermore, ADSCs-Exos could block the increase in ROS content and MDA level in HG-stimulated MPC5 cells, but HO-1 siRNA could block this regulatory effect (Fig. 3E F). Taken together, we concluded that ADSCs-Exos ameliorated the HG-induced oxidative stress and inflammation by reversing the decrease in HO-1 expression.

### ADSCs-Exos circumvent Nrf2 pathway inhibition in HG-stimulated podocytes

**Fig. 4:**
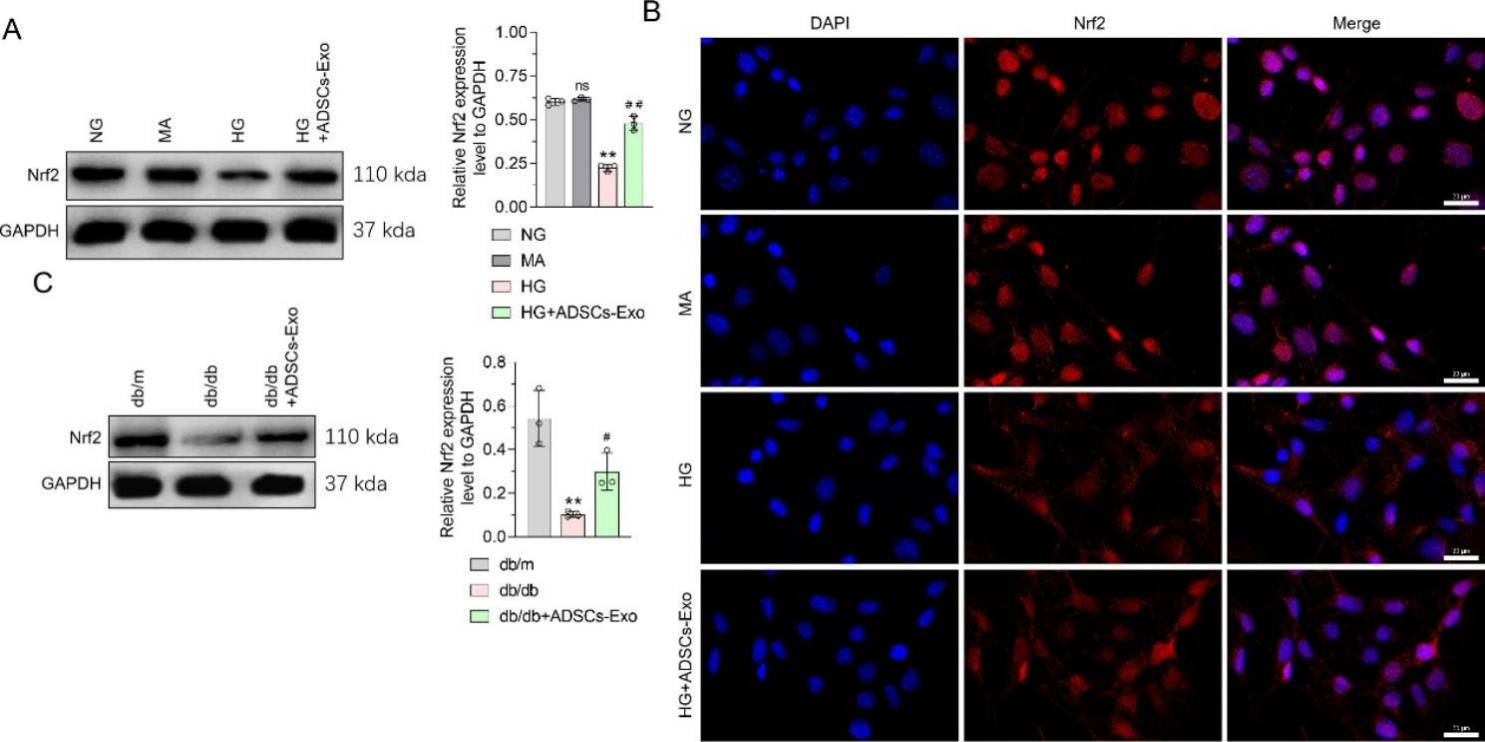
**Effects of ADSCs-Exo and high glucose treatment on Nrf2 pathway of podocytes** **(A)** WB was used to detect the effect of ADSCs-Exo and high glucose on Nrf2 expression in MPC5 cells, and GAPDH was used as an internal control. **(B)** IF was used to detect the effects of ADSCs-Exo and high glucose treatment on Nrf2 distribution in MPC5 nucleus; **(C)** WB was used to detect the effect of ADSCs-Exo on Nrf2 expression in kidney tissue of model mice, and GAPDH was used as an internal control. Data are presented as Mean ± SD and one-way analysis of variance was used to detect statistical differences between groups. Panel A. ns p > 0.05 VS. NG; ** p < 0.01 VS. NG; ## p < 0.01VS. HG. Panel C.** p < 0.01 VS. db/m; # p < 0.05 VS. db/db. The scale in Panel B is 20 μm

As HO-1 is a downstream regulator of Nrf2, we investigated on the effects of HG and ADSC-Exo treatment on Nrf2 content in podocytes. As shown in Fig. 4A, the total Nrf2 level in MPC5 cells was similar in the MA and NG groups, but was greatly reduced in the HG group. Nrf2 expression in MPC5 cells was significantly higher in the ADSC-Exo treatment group than in the HG treatment group (Fig. 4A). Immunofluorescence showed the same expression trend and further demonstrated that HG stimulation could reduce the Nrf2 content in the nucleus of MPC5 cells, compared with the NG group. After ADSC-Exo treatment, the reduction in Nrf2 levels in the MPC5 nucleus was alleviated in the HG group (Fig. 4B).

The in vivo experimental results showed that Nrf2 expression in the kidney tissue of DN mice was significantly lower than that in the kidney tissue of db/m mice. Additionally, DN mice treated with ADSCs-Exos showed higher Nrf2 expression in renal tissues than untreated controls (Fig. 4C). Thus, ADSCs-Exos reversed the HG-induced oxidative stress, podocyte inflammation, and decreased HO-1 expression, which may be related to the reactivation of Nrf2 signaling.

### ADSCs-Exos inhibit the increased Keap1 expression and increased Keap1-Nrf2 binding capacity in HG-stimulated podocytes

**Fig. 5:**
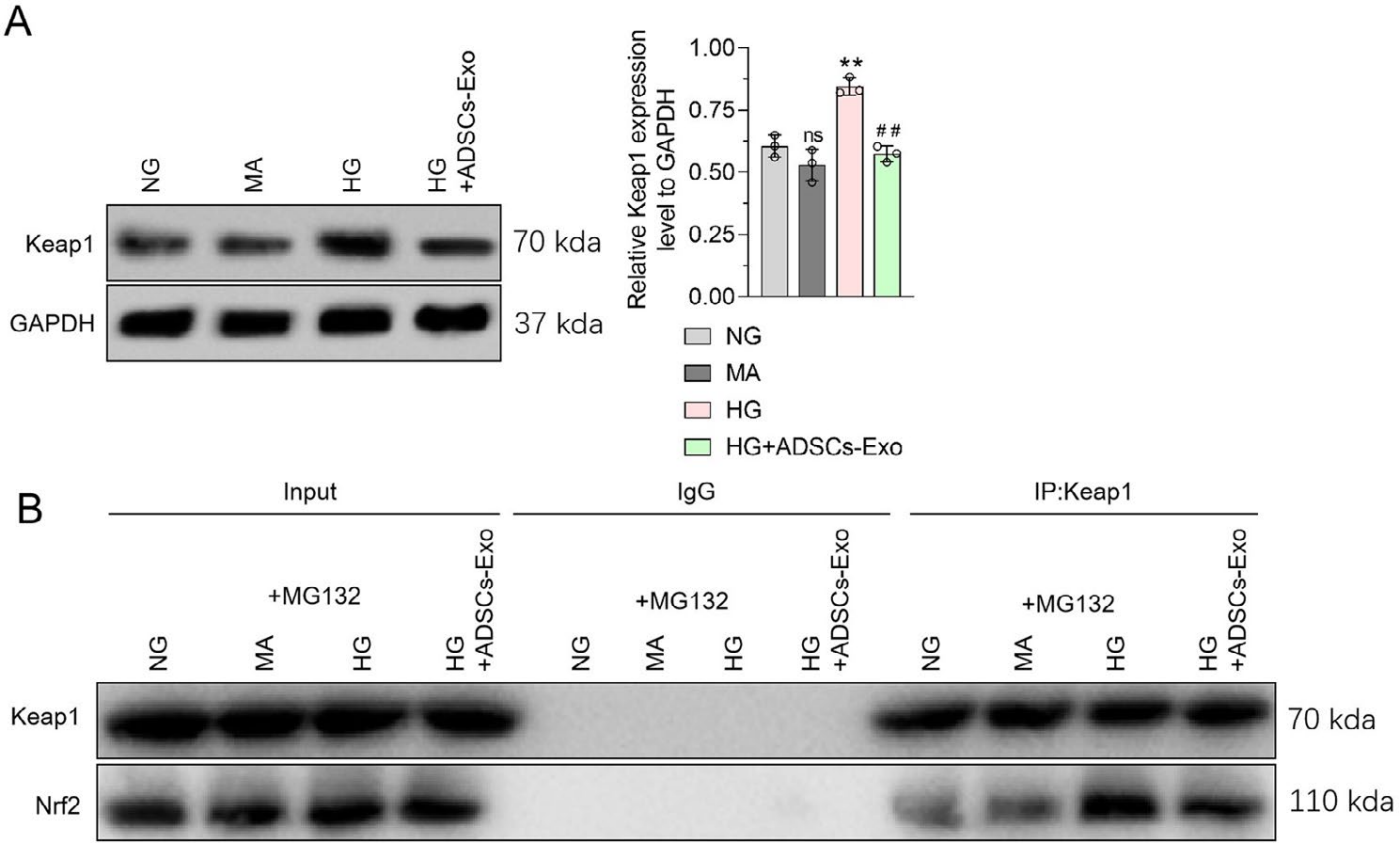
**Effects of ADSCs-Exo and high glucose treatment on Keap1 expression and Keap1-NrF2 binding in podocytes** **(A)** WB was used to detect the effect of ADSCs-Exo and high glucose treatment on Keap1 protein expression in MPC5 cells, and GAPDH was used as an internal control. **(B)** CO-IP was used to detect the effect of ADSCs-Exo and high glucose on Keap1-Nrf2 binding in MPC5 cells, with input as the internal control. Data were presented as Mean ± SD, and one-way analysis of variance was used to detect statistical differences between groups. ns p > 0.05 VS. NG; ** p < 0.01 VS. NG; ## p < 0.01 VS. HG.

Nrf2 binding to Keap1 can increase Nrf2 ubiquitination, promote its degradation through the ubiquitin-protein enzyme pathway, and ultimately inhibit its expression. Western blotting showed no significant difference in the MPC5-cell Keap1 expression in the MA group, compared with the low-glucose treatment group, whereas significantly increased Keap1 expression was observed in the HG group. In ADSCs-Exos-treated cells, Keap1 expression was significantly lower than that in HG-treated cells (Fig. 5A). To better observe the binding ability changes in Keap1 and Nrf2, we treated MPC5 cells with MG132 to block the Nrf2 ubiquitin-proteasome degradation pathway. As shown in the input images, MG132 eliminated the regulatory effects of HG and ADSCs-Exos on Nrf2 expression in MPC5 cells. It is suggested that HG and ADSCs-Exos affect Nrf2 ubiquitin-proteasome degradation in podocytes. Additionally, MG132 reversed the HG and ADSC-Exo treatment-induced changes in Keap1 expression in MPC5 cells. Accordingly, the ubiquitin-proteasome degradation pathway may be involved in changes in Keap1 expression in HG and ADSCs-Exos-stimulated podocytes. Keap1 and Nrf2 were not detected in the IgG antibody CO-IP group (negative control), while Keap1 and Nrf2 proteins were present in Keap1 antibody CO-IP group (Fig. 5B), indicating that Keap1 could bind to Nrf2 in podocytes. Furthermore, compared with the negative control, the binding of Keap1 and Nrf2 in MPC5 cells did not change in the MA group, but increased in the HG group. Intracellular Keap1 and Nrf2 binding decreased in the ADSCs-Exos group. Taken together, our results indicate that ADSCs-Exos could reverse the promoting effect of HG on Keap1 expression and the ability of Keap1 to bind to Nrf2 proteins in podocytes.

**ADSCs-Exos improve oxidative stress and inflammation by inhibiting the HG-induced reduction of FAM129B in podocytes and thereby activating the Nrf2 pathway**.

**Fig. 6:**
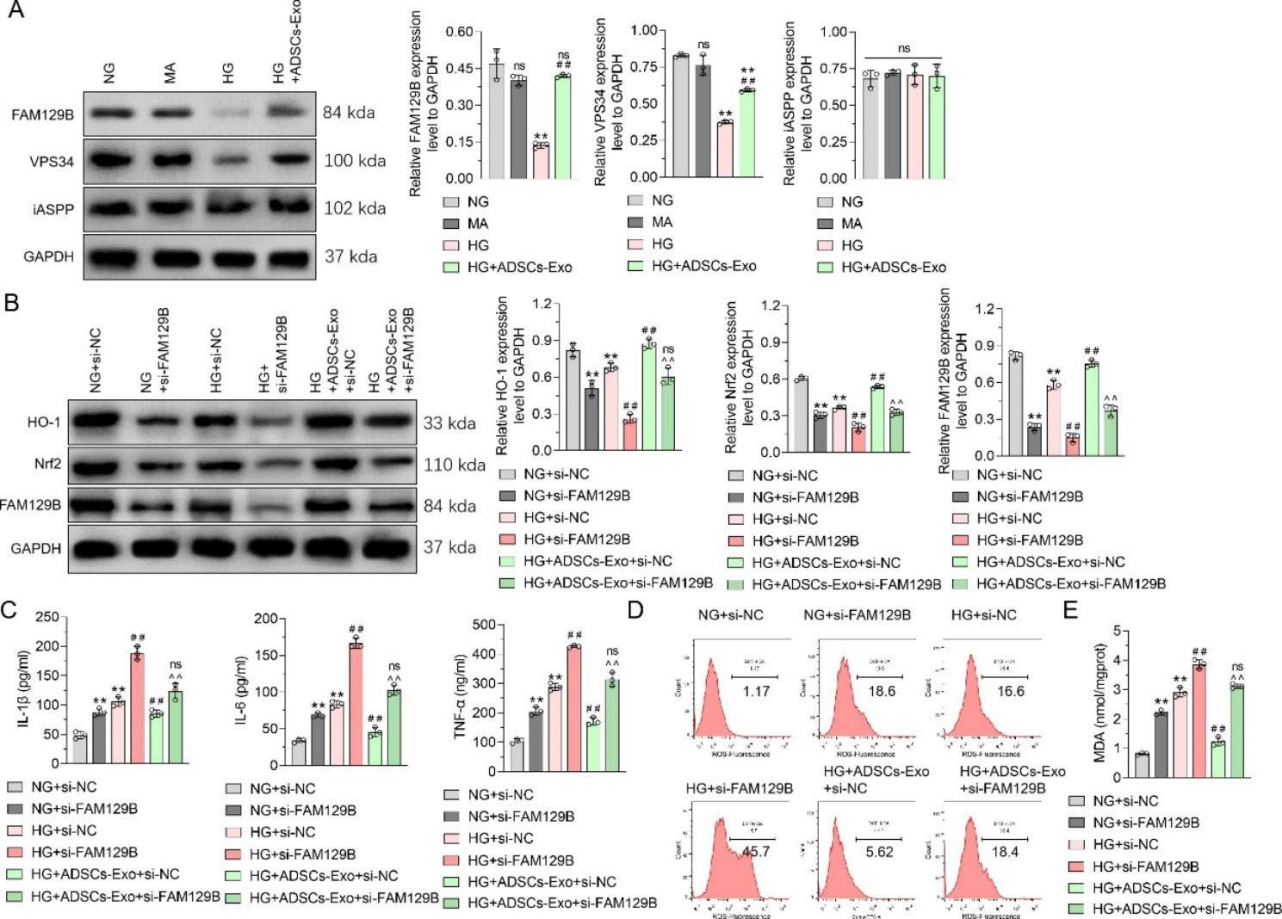
**Effects of FAM129B and ADSCs-Exo on Nrf2 and HO-1 expression, pro-inflammatory cytokine secretion, and oxidative stress in podocytes treated with high glucose** **(A)** WB was used to detect the effects of ADSCs-Exo and high glucose treatment on FAM129B, VPS34, and iASPP expression in MPC5 cells, with GAPDH as an internal control. **(B)** WB was used to detect the effects of FAM129B siRNA and ADSCs-Exo on HO-1, Nrf2, and FAM129B expression in MPC5 cells treated with high glucose, with GAPDH as an internal control. **(C)** ELISA was used to detect the effect of FAM129B siRNA and ADSCs-Exo on the secretion of pro-inflammatory factors in MPC5 cells under high glucose treatment. **(D)** Flow cytometry was used to detect the effects of FAM129B siRNA and ADSCs-Exo on intracellular ROS content in MPC5 cells under high glucose treatment. **(E)** MDA kit was used to detect the effects of FAM129B siRNA and ADSCs-Exo on MDA content in MPC5 cells treated with high glucose. Data are presented as Mean ± SD and one-way analysis of variance was used to detect statistical differences between groups. Panel A, ns p > 0.05 VS. NG; **p < 0.01VS. NG; ##p < 0.01 VS. HG. Panel B, C, E, ** p < 0.01 VS. NG + si-NC; ns p > 0.05, ## p < 0.01 VS. HG + si-NC; ^^ p < 0.01 VS. HG + ADSCs-Exo + si-NC.

Next, we explored the specific mechanism, by which ADSCs-Exos regulate the Nrf2 pathway. Literature search revealed that FAM129B [25], VPS34 [26] and iASPP [27] can competitively bind to Keap1, inhibiting Keap1-Nrf2 binding to activate Nrf2. Therefore, we investigated the effects of HG and ADSC-Exo treatment on FAM129B, VPS34, and iASPP levels in MPC5 cells using western blotting. MPC5 cells in the HG group had lower FAM129B and VPS34 contents than those in the NG group, but not iASPP. Notably, the reduction in FAM129B expression was greater than that of VPS34 under HG treatment. MPC5 cells treated with ADSCs-Exos had significantly higher FAM129B and VPS34 contents than those treated with HG, and ADSCs-Exos completely blocked the inhibitory effect of HG on FAM129B (Fig. 6A). In the following study, si-FAM129B-1 was selected as the most effective siRNA to inhibit FAM129B protein expression in MPC5 cells, according to western blot analysis (Supplementary Fig. 2). The reverse effect of ADSCs-Exos on decreased FAM129B expression in HG-stimulated MPC5 cells was blocked by FAM129B siRNA. In addition, FAM129B siRNA reduced Nrf2 and HO-1 levels in MPC5 cells and inhibited ADSCs-Exos by promoting Nrf2 expression in HG-treated MPC5 cells (Fig. 6B). The secretion of inflammatory factors detected using ELISA showed that FAM129B siRNA increased IL-1β, IL-6, and TNF-α secretion in MPC5 cells in the NG group and intensified their secretion in the HG group. ADSCs-Exos also blocked the inhibition of IL-1β, IL-6, and TNF-α secretion in HG-stimulated MPC5 cells (Fig. 6C). Moreover, FAM129B siRNA increased intracellular ROS and MDA levels, regardless of whether the MPC5 cells were stimulated through hyperglycemia. FAM129B siRNA blocked the inhibitory effect of ADSCs-Exos on ROS and MDA upregulation in HG-treated MPC5 cells (Fig. 6D and E). Altogether, the effects of ADSCs-Exos at reducing Nrf2 and HO-1 expression in podocytes, as well as reducing oxidative stress and inflammatory cytokine secretion all depend on FAM129B.

## Discussion

The prevalence of DN has been increasing year by year and has become one of the leading causes of end-stage renal disease (ESRD) [28]. At present, the treatment of diabetic nephropathy is mainly through symptomatic treatment such as lowering blood sugar, controlling blood pressure and reducing proteinuria. There is still a lack of effective targeted therapeutic drugs for diabetic nephropathy [2]. Accumulating studies have shown that MSCs play a role in promoting tissue regeneration [29], immune regulation [3], anti-inflammation [30], pro-angiogenesis [31], and anti-apoptosis [32] through cell-cell interactions and the secretion of growth factors, cytokines, chemokines, cell adhesion molecules, lipid mediators, hormones, exosomes, microvesicles, and other regulatory molecules, such as miRNA, which have demonstrated a potential therapeutic role in a variety of diseases [33–35]. ADSCs-Exo can ameliorate HG-induced podocyte injury and DN progression, mainly through the mir-486-Smad2-autophagy and the miR-215-5p-ZEB2 signal transduction pathways [24]. Here, we showed that ADSCs-Exos could attenuate HG-induced inflammatory cytokine secretion and lipid peroxidation in vitro and in vivo.

Inflammatory factors induce podocyte injury and promote DN progression through nuclear factor kappa B, toll-like receptors, and adenosine 5’-monophosphate-activated protein kinase signaling pathways [36]. Stem cell-derived exosomes have the potential to ameliorate the destructive effects of inflammatory factors and alleviate tissue damage, suggesting that the effect of ADSCs-Exo on podocyte injury and DN progression may be closely related to its anti-inflammatory and antioxidant functions [37]. We have previously found that HO-1 can inhibit podocyte apoptosis by up-regulating autophagy level [38]. It has also been reported that up-regulation of Nrf2/HO-1 signaling can induce mitochondrial autophagy, oxidative stress, inflammation, apoptosis and angiogenesis in diabetic nephropathy rats[39–42]. Here, we found that HG significantly reduced HO-1 expression in MPC5 cells, which was partially reversed by ADSCs-Exo treatment. Our results showed that inhibiting HO-1 expression reversed the anti-inflammatory and anti-oxidative effects of ADSCs-Exos on podocytes, confirming that ADSCs-Exo could improve HG-induced podocyte inflammation and oxidative stress by up-regulating HO-1 expression.

As a key regulatory pathway for HO-1, Keap1/Nrf2/ARE regulates the transcription of many antioxidant genes under stress-inducing, inflammatory, and pro-apoptotic conditions [43]. Liu et al. [44] found that Nrf2 deficiency exacerbates diabetic kidney disease in compound mutant mice. Exosomes can exert anti-inflammatory and anti-oxidative effects by regulating the Nrf2 pathway [45, 46]. We showed that ADSCs-Exos could reverse HG-induced Nrf2 reduction in vitro and in vivo, indicating that ADSCs-Exo can regulate high glucose-induced HO-1 dependent anti-inflammatory and anti-oxidative function in podocytes, which is related to the up-regulation of Nrf2 expression. Nrf2 binds to Keap1 under physiological conditions, resulting in increased ubiquitination on Nrf2 and promoting Nrf2 degradation through the ubiquitin-proteasome pathway [47, 48]. Under oxidative stress, Nrf2 was transported to the nucleus and involved in the transcription of a series of antioxidant genes [49]. We found that Nrf2 content in podocytes was not altered by HG-stimulation under MG132 treatment. However, Nrf2 expression in podocytes decreased, while Keap1 expression was increased in podocytes under HG treatment. It is speculated that HG promotes the ubiquitination of Nrf2 by increasing Keap1 expression, which mediates Nrf2 degradation through the ubiquitin-proteasome pathway, and eventually leads to the reduction of Nrf2 content in podocytes. This may be the reason why HG reduces intracellular Nrf2 expression despite activating oxidative stress in podocytes. In addition, ADSCs-Exo reduced Keap1 expression in podocytes under high glucose treatment, but this regulatory effect was abolished under MG132 treatment. These results indicated that ADSCs-Exo could reduce Keap1 content and block Keap1-mediated Nrf2 degradation, which eventually led to Nrf2 accumulation in podocytes under high glucose treatment. Interestingly, Co-IP showed that ADSCs-Exos reversed the HG-induced promotion of Keap1 and Nrf2 binding in podocytes.

FAM129B is an antioxidant protein with anti-apoptotic effects in tumors, which can compete with Nrf2 and bind Keap1 to reduce Nrf2 ubiquitination, so as to activate the Nrf2 pathway [25, 50]. Here, FAM129B expression decreased in HG-stimulated podocytes, and FAM129B knock-down enhanced HO-1 and Nrf2 expression, inflammatory factor secretion, and ROS content in podocytes. This suggests that HG could inhibit FAM129B expression, block the binding between FAM129B and Keap1, and enhance the binding between Keap1 and Nrf2, resulting in the reduction of Nrf2 expression and the impairment of HO-1 mediated anti-inflammatory and antioxidant functions. ADSCs-Exo significantly reversed the inhibition of FAM129B expression in high glucose-stimulated podocytes. In addition, the inhibitory effect of ADSCs-Exo on the decrease of HO-1 and Nrf2 expression and the increase of inflammatory factor secretion and ROS content in HG-stimulated podocytes could be reversed by FAM129B knock-down.

However, as ADSCs-Exos are mainly carriers of active components, it is unclear what specific compounds they contained. Detailed studies are needed to clarify the specific components of ADSCs-Exos that inhibit inflammation and oxidative stress.

## Conclusion

This study clarified that ADSCs-Exos have exhibited a therapeutic role in DN. ADSCs-Exos relieve HG-induced oxidative stress and inflammation in podocytes by upregulating FAM129B and reactivating the Nrf2-HO-1 pathway, thus providing a new perspective for the clinic.

## Electronic supplementary material

Below is the link to the electronic supplementary material.

Supplementary Material 1

Supplementary Material 2

Supplementary Material 3

## Data Availability

The datasets generated during the current study are available from the corresponding author on reasonable request.
