# Supplementary material for: Adipose-derived stem cell exosomes regulate Nrf2/Keap1 in diabetic nephropathy by targeting FAM129B
Source: Diabetol Metab Syndr. 2023 Jul 4;15:149. doi: 10.1186/s13098-023-01119-5 (PMC10318792; doi:10.1186/s13098-023-01119-5)
Supplement: Supplementary file 1 — Supplementary Material 1 [file 13098_2023_1119_MOESM1_ESM.docx]

# Supplement

**Supplementary Table 1: siRNA sequences**

| siRNA | Sense sequences (5′–3′) | Anti-sense sequences (5′–3′) |  |
| --- | --- | --- | --- |
| HO-1 siRNA1 | CCAAGUUCAAACAGCUCUAUC | UAGAGCUGUUUGAACUUGGUG |  |
| HO-1 siRNA2 | GGGUGACAGAAGAGGCUAAGA | UUAGCCUCUUCUGUCACCCUG |  |
| HO-1 siRNA3 | GGUGAUGCUGACAGAGGAACA | UUCCUCUGUCAGCAUCACCUG |  |
| FAM129B siRNA1 | GGUGCUAUAUGAGAACAAAGU | UUUGUUCUCAUAUAGCACCAG |  |
| FAM129B siRNA2 | GGUUCAUCCUGGUGGAGAACA | UUCUCCACCAGGAUGAACCUG |  |
| FAM129B siRNA3 | GAUCCACAUGCGAGAGCAAAU | UUGCUCUCGCAUGUGGAUCUG |  |
| NC siRNA | GAAUUAAUUAAAGAUGGCCCGUUGUACU | UCAUCGAAGUUAUAGGGAUACAUUACGUGAUC |  |

**Supplementary Table 2: Antibody information**

| **Antibody** | **Supplier** | **Catalog Number** | **dilutions** | **Applications** |
| --- | --- | --- | --- | --- |
| anti-HO-1 | Abcam | ab52947 | 1:1000 | WB |
| anti-Keap1 | Abcam | ab227828 | 1:1000 | WB |
| anti-Nrf2 | Abcam | ab137550 | 1:1000 | WB |
| anti-FAM129B | Abcam | ab224116 | 1:1000 | WB |
| anti-VPS34 | Abcam | ab124905 | 1:1000 | WB |
| anti-iASPP | Abcam | ab34898 | 1:1000 | WB |
| anti- GAPDH | Abcam | ab8245 | 1:1000 | WB |
| anti IgG | CST | 3423 | 1:50 | IP |
| anti-Keap1 | CST | 4678 | 1:50 | IP |
| NRF2 | Proteintech | 16396-1-AP | 1:100 | IF |
| goat anti-rabbit IgG (H+L) | affinity | S0001 | 1:5000 | WB, IP |
