# Supplementary material for: Adipose-derived stem cell exosomes regulate Nrf2/Keap1 in diabetic nephropathy by targeting FAM129B
Source: Diabetol Metab Syndr. 2023 Jul 4;15:149. doi: 10.1186/s13098-023-01119-5 (PMC10318792; doi:10.1186/s13098-023-01119-5)
Supplement: Supplementary file 2 — Supplementary Material 2 [file 13098_2023_1119_MOESM2_ESM.pptx]

## Slide 1
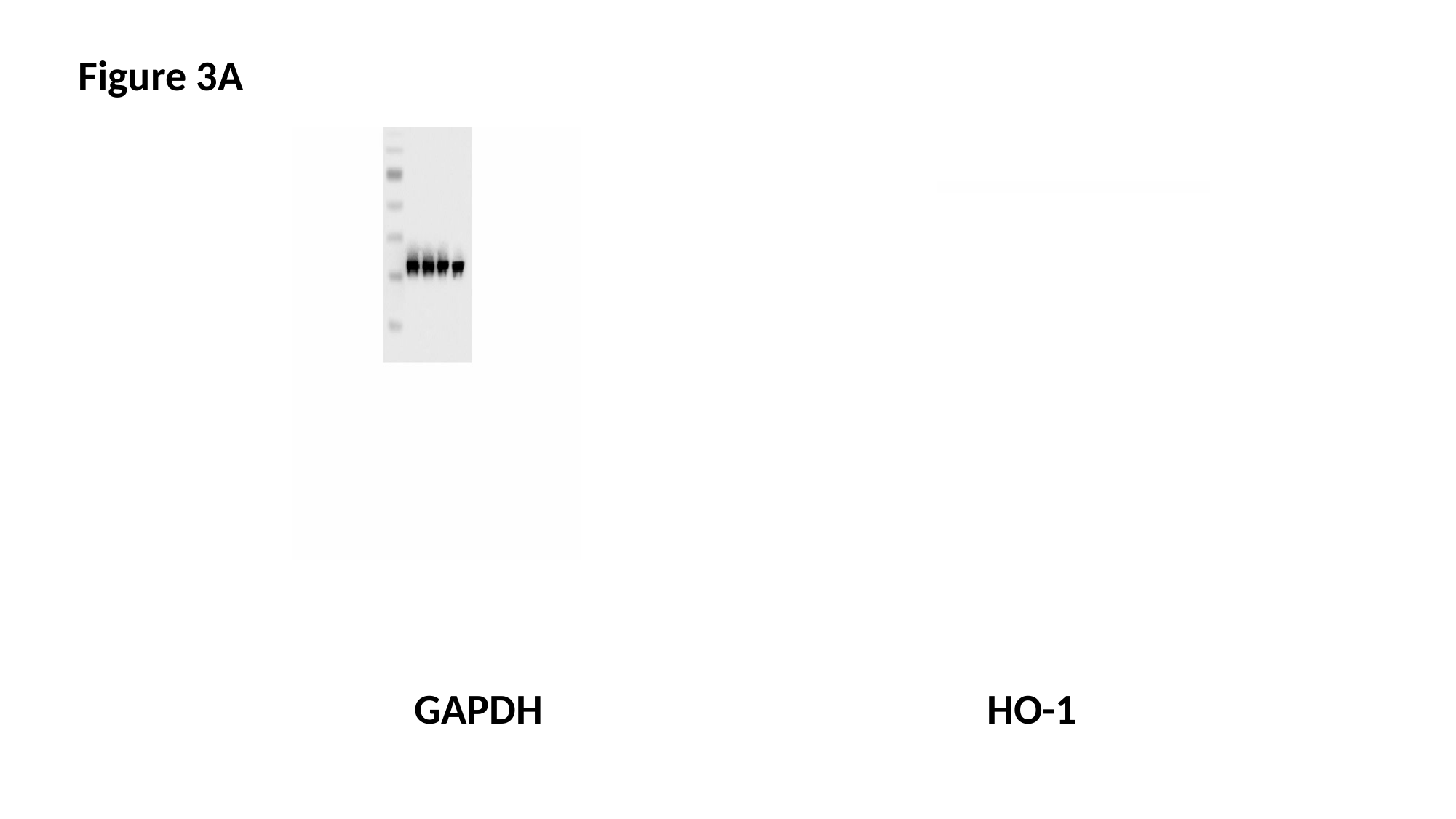

Figure 3A
GAPDH
HO-1

## Slide 2
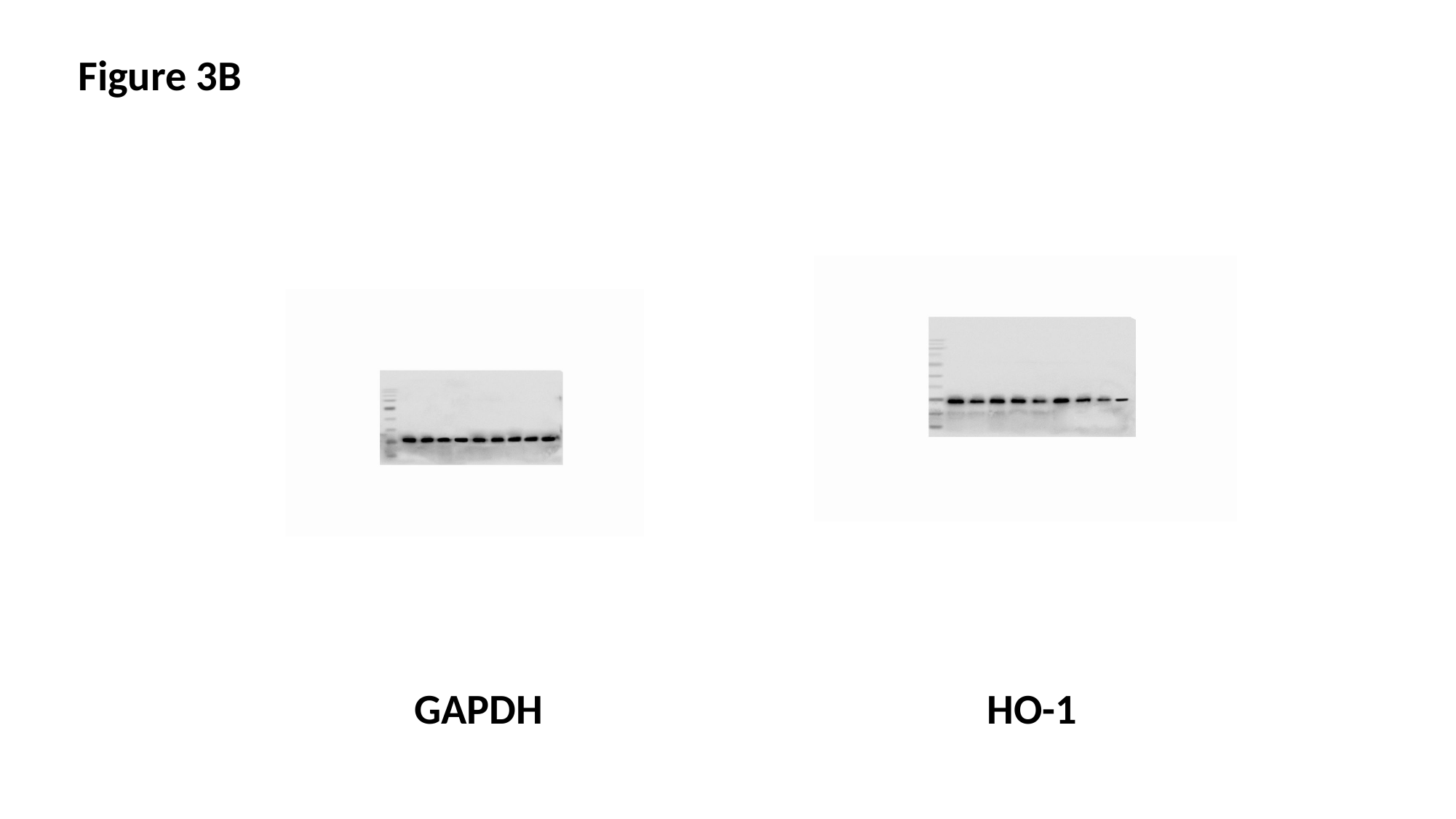

Figure 3B
GAPDH
HO-1

## Slide 3
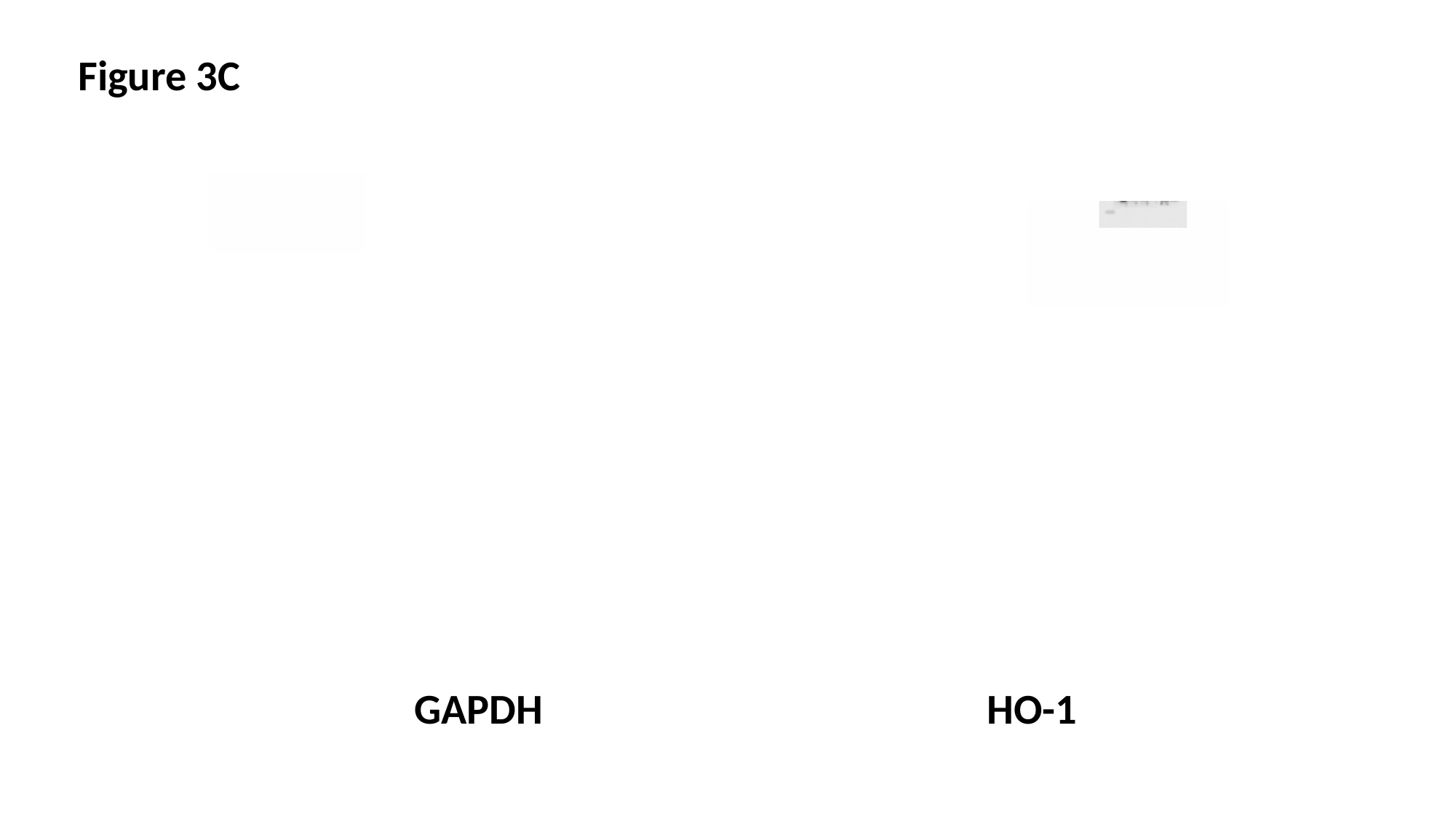

Figure 3C
GAPDH
HO-1

## Slide 4
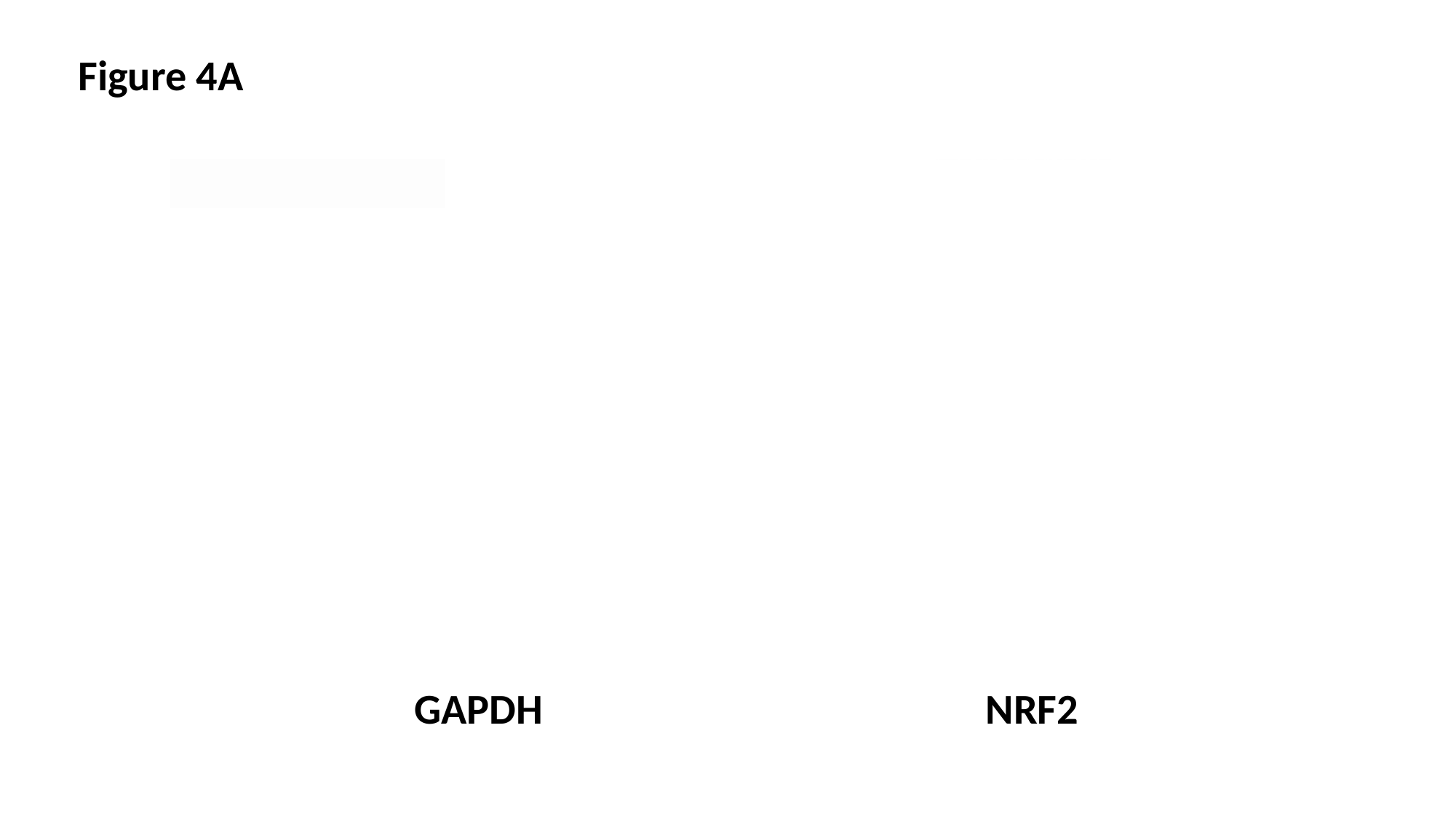

Figure 4A
GAPDH
NRF2

## Slide 5
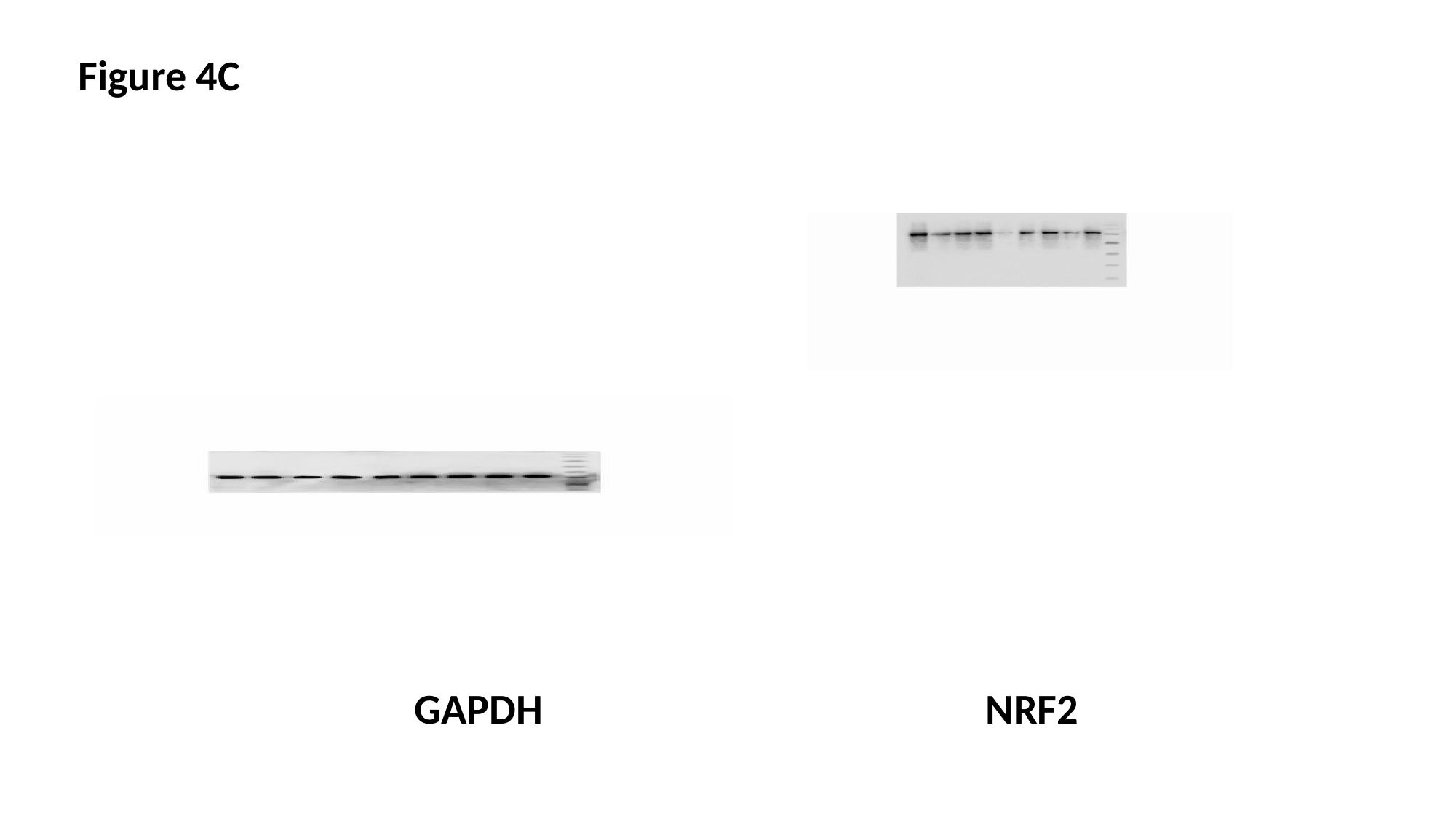

Figure 4C
GAPDH
NRF2

## Slide 6
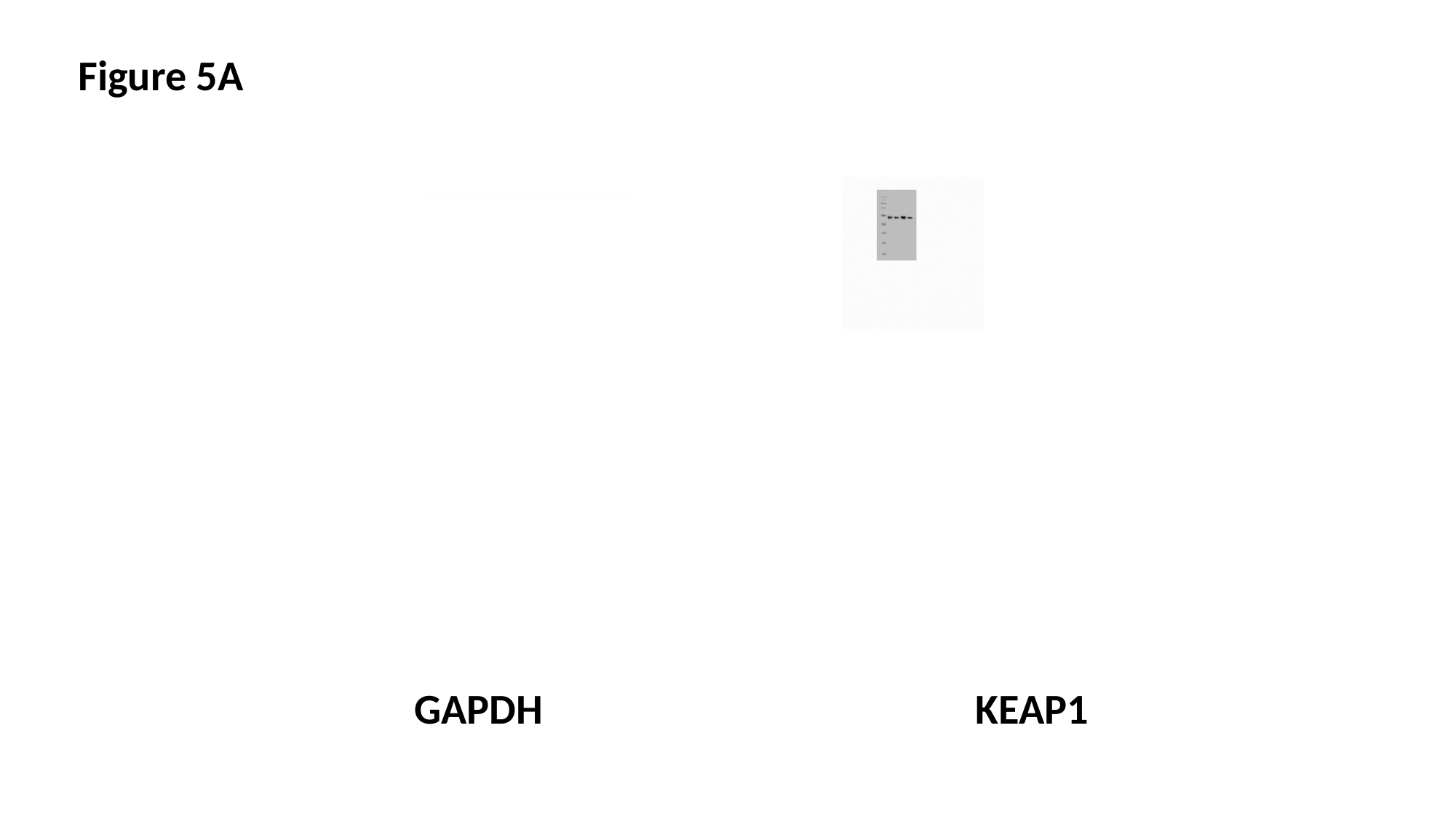

Figure 5A
GAPDH
KEAP1

## Slide 7
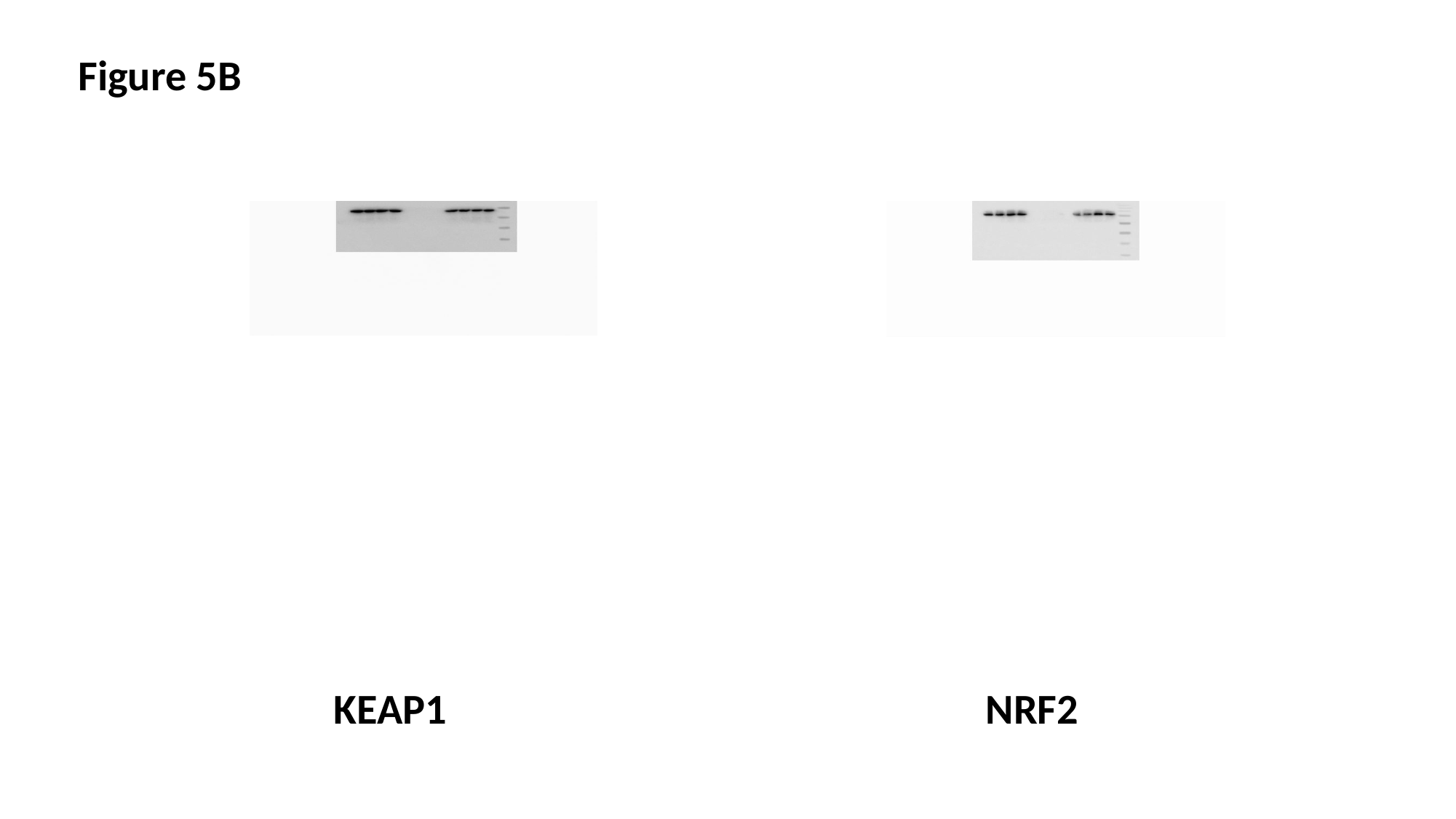

Figure 5B
KEAP1
NRF2

## Slide 8
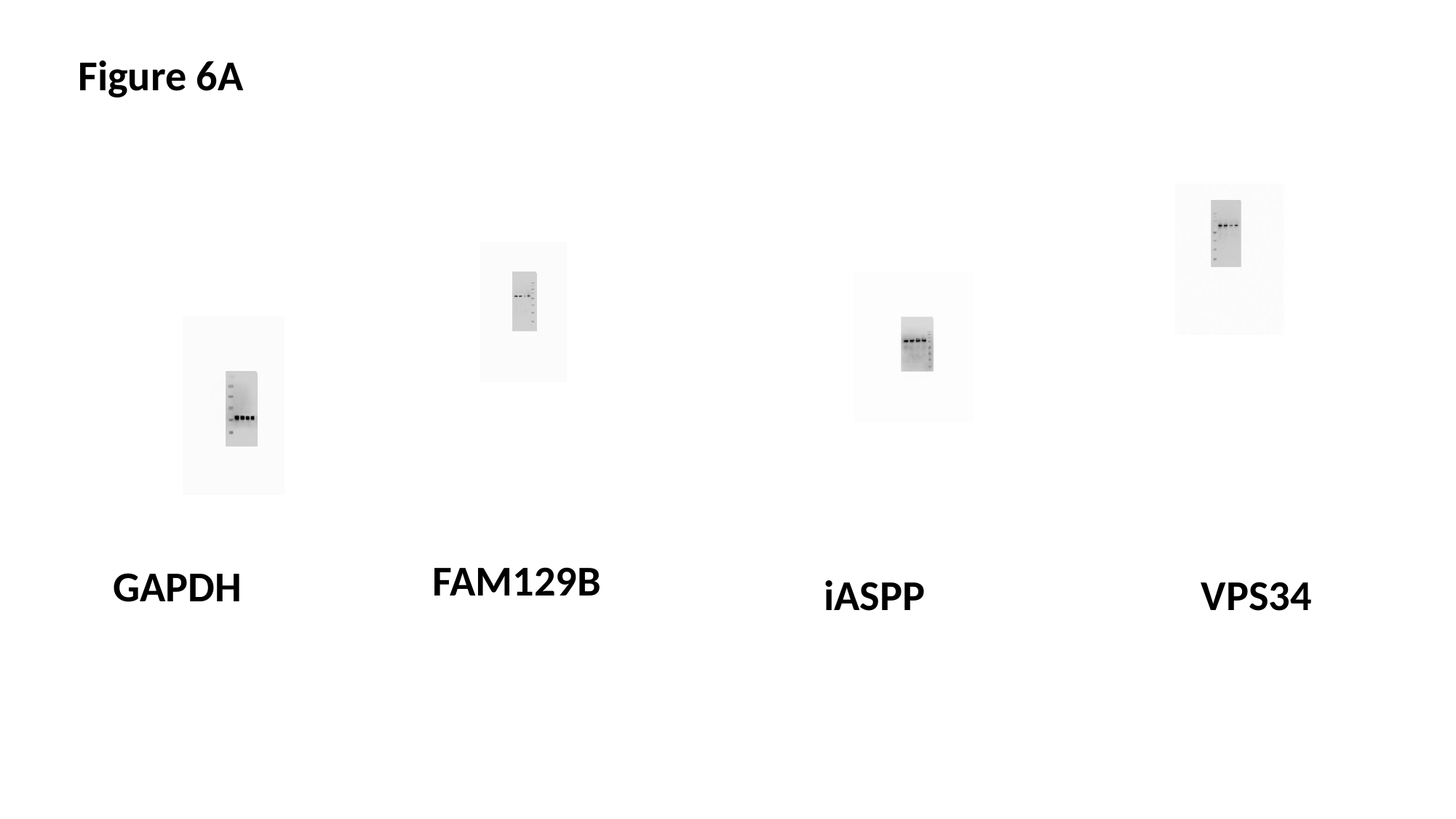

Figure 6A
GAPDH
FAM129B
iASPP
VPS34

## Slide 9
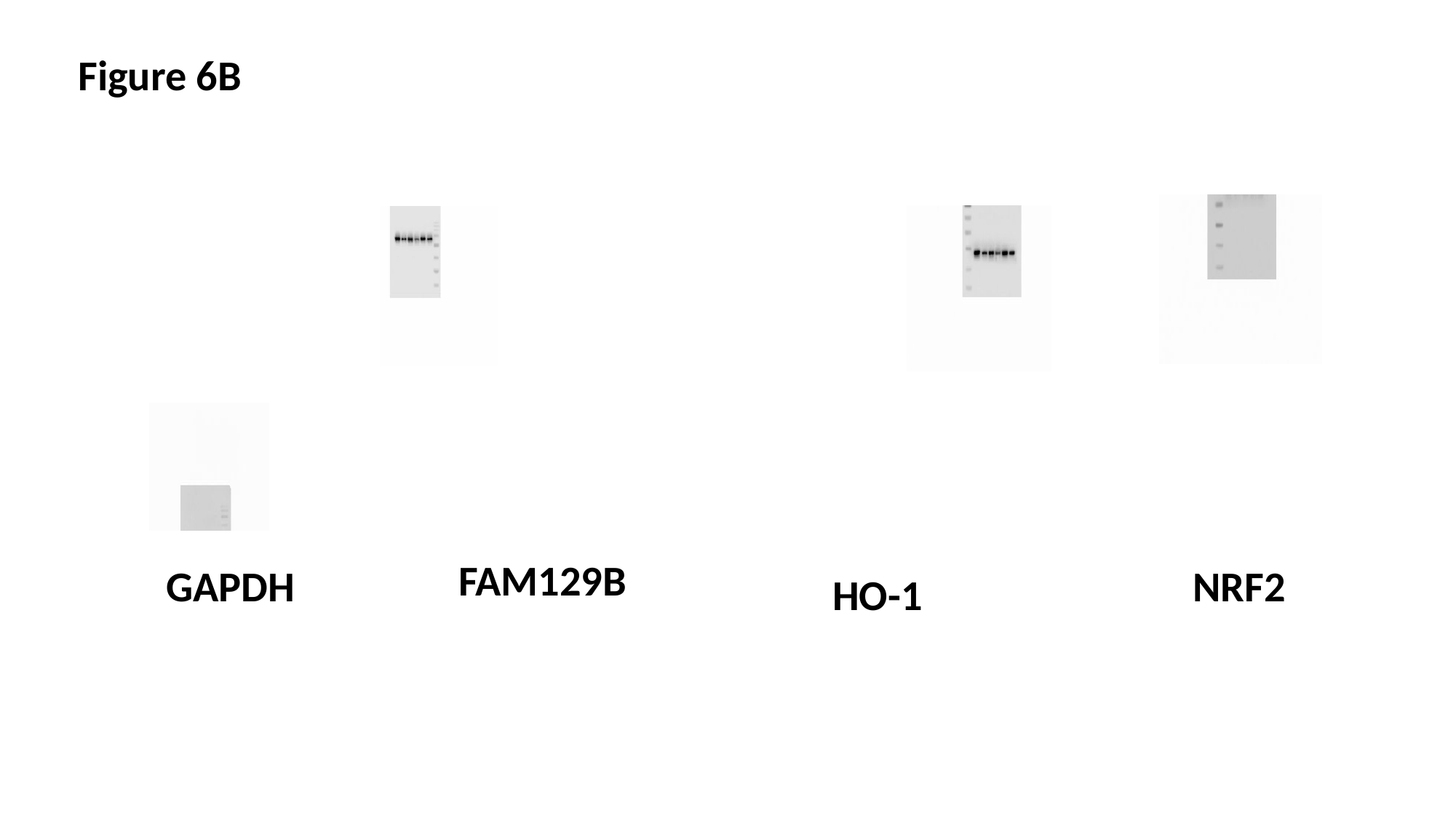

Figure 6B
FAM129B
GAPDH
NRF2
HO-1
