# Supplementary material for: Adipose-derived stem cell exosomes regulate Nrf2/Keap1 in diabetic nephropathy by targeting FAM129B
Source: Diabetol Metab Syndr. 2023 Jul 4;15:149. doi: 10.1186/s13098-023-01119-5 (PMC10318792; doi:10.1186/s13098-023-01119-5)
Supplement: Supplementary file 3 — Supplementary Material 3 [file 13098_2023_1119_MOESM3_ESM.docx]

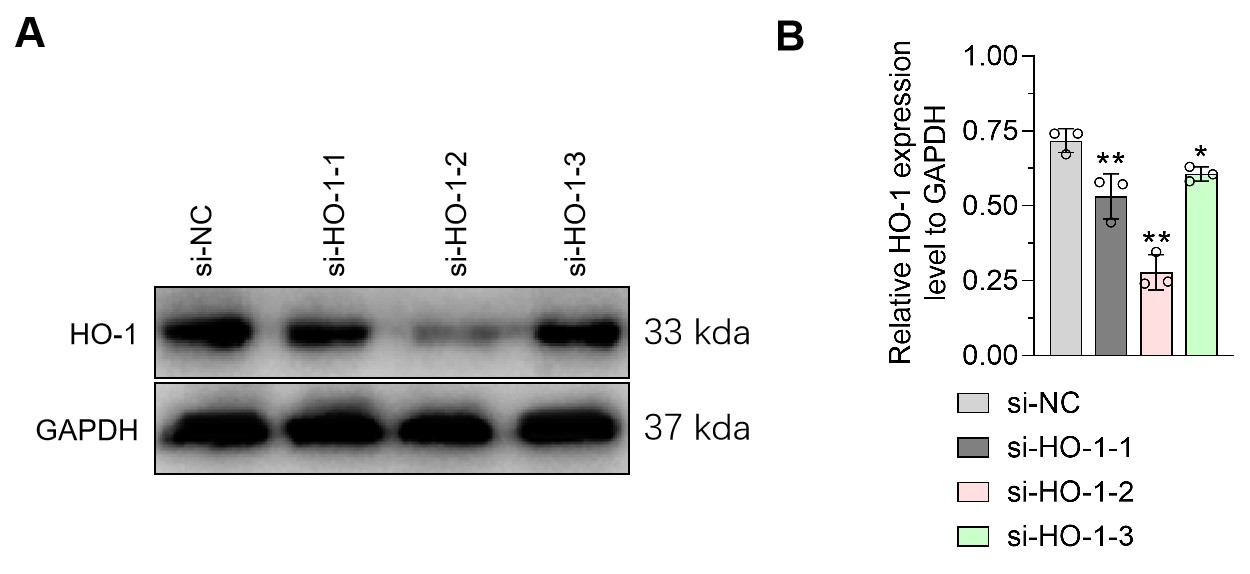


**Supplementary figure 1: Effect of HO-1 siRNA on HO-1 expression in podocytes.**

**A.** The effect of HO-1 siRNA on HO-1 protein content in MPC5 cells was detected by WB, GAPDH was used as an internal control. **B.** Statistical chart of gray value of strips in A; Data are presented as Mean± SD and one-way analysis of variance was used to detect statistical differences between groups. * p<0.05, ** p<0.01 VS. si-NC.


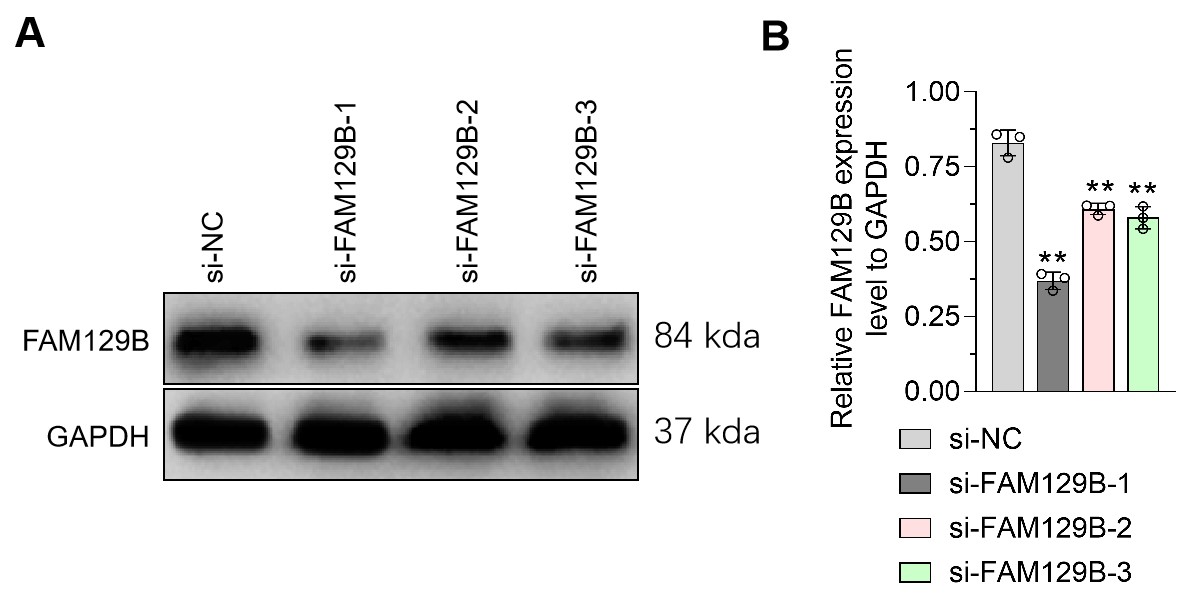


**Supplementary Figure2. Effect of FAM129B siRNA on FAM129B expression in podocytes.**

**A.** WB was used to detect the effect of FAM129B siRNA on FAM129B protein content in MPC5 cells, with GAPDH as an internal control. **B.** Statistical chart of gray value of strips in A; Data are presented as Mean± SD and one-way analysis of variance was used to detect statistical differences between groups. ** p<0.01 VS. si-NC.


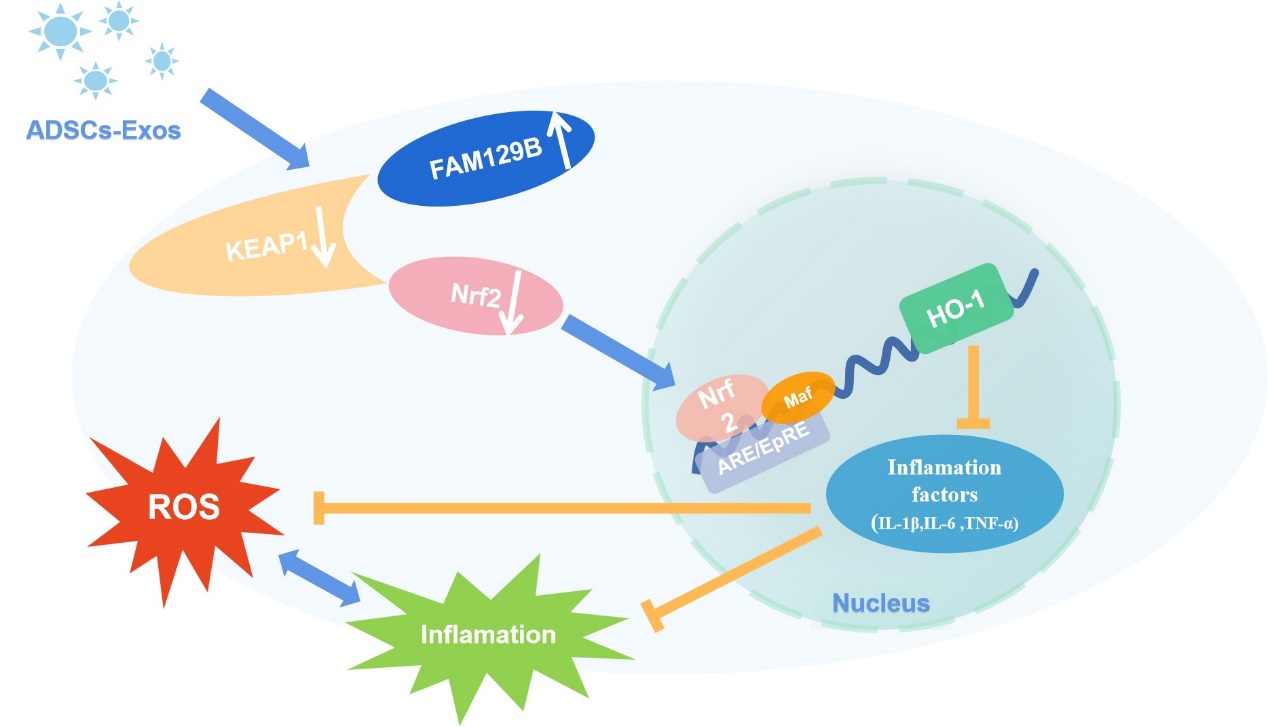


**Summary schematic diagram：ADSCs-Exos relieve HG-induced oxidative stress and inflammation in podocytes by upregulating FAM129B and reactivating the Nrf2-HO-1 pathway**
